# Cross-Coupling Reactions as Valuable Tool for the Preparation of PET Radiotracers

**DOI:** 10.3390/molecules16021129

**Published:** 2011-01-26

**Authors:** Marc Pretze, Philipp Große-Gehling, Constantin Mamat

**Affiliations:** 1 Institut für Radiopharmazie, Helmholtz-Zentrum Dresden-Rossendorf, Bautzner Landstraße 400, D-01328 Dresden, Germany; 2 OncoRay – National Center for Radiation Research in Oncology, Medical Faculty Carl Gustav Carus, Dresden University of Technology, Fetscherstraße 74, P.O. Box 41, D-01307 Dresden, Germany

**Keywords:** cross-coupling, radiolabeling, carbon-11, fluorine-18

## Abstract

The increasing application of positron emission tomography (PET) in nuclear medicine has stimulated the extensive development of a multitude of new radiotracers and novel radiolabeling procedures with the most prominent short-lived positron emitters carbon-11 and fluorine-18. Radiolabeling with these radionuclides represents a remarkable challenge. Special attention has to be paid to synthesis time and specific labeling techniques due to the short physical half life of the respective radionuclides ^11^C (t_1/2_ = 20.4 min) and ^18^F (t_1/2_ = 109.8 min). In the past, numerous transition metal-catalyzed reactions were employed in organic chemistry, even though only a handful of these coupling reactions were adopted in radiochemical practice. Thus, the implementation of modern synthesis methods like cross-coupling reactions offers the possibility to develop a wide variety of novel radiotracers. The introduction of catalysts based on transition metal complexes bears a high potential for rapid, efficient, highly selective and functional group-tolerating incorporation of carbon-11 and fluorine-18 into target molecules. This review deals with design, application and improvement of transition metal-mediated carbon-carbon as well as carbon-heteroatom cross-coupling reactions as a labeling feature with the focus on the preparation of radiolabeled compounds for molecular imaging.

## 1. Introduction

Radiopharmaceutical chemistry deals with the design and synthesis of radiolabeled compounds, also referred to as radiotracers. These days, radiopharmaceutical chemistry has developed into a complex chemical science which combines progress in modern organic and inorganic chemistry with novel trends in molecular biology. Development of new radiotracers for molecular imaging has to account for special requirements of their preparation with respect to the choice of the appropriate radionuclide and the labeling position. Furthermore, the field of radiotracers ranges from small organic and bioactive molecules such as carbohydrates, amino acids or steroids to high molecular weight compounds like peptides, proteins or oligonucleotides. Therefore, special attention should be paid to the implementation of fast and highly selective reactions which tolerate other functional groups. As enzyme and transition metal-catalyzed reactions both comply with these requirements, they were introduced for the design and synthesis of a multitude of radiotracers labeled with carbon-11 (t_1/2_ = 20.4 min) and fluorine-18 (t_1/2_ = 109.8 min). The catalyst in cross-coupling reactions applied for radiolabeling purposes is used in large excess compared to the respective carbon-11 or fluorine-18- containing building block. Due to this unusual stochiometrical scale, the term "mediated" is rather appropriate than "catalyzed", as it is statistically unlikely for the metal to take part in another cycle. Therefore, the term “metal-mediated” is preferred in radiolabeling reactions. 

Transition metal-catalyzed cross-coupling reactions have been used extensively in carbon-carbon as well as carbon-heteroatom bond-forming reactions [1]. The central importance of these reactions using cross-coupling methods in organic chemistry as well as life sciences was honored with the Nobel Price in Chemistry in the year 2010 [2]. In the majority of cases, this kind of reactions is adopted for the preparation of the precursor scaffold which is subsequently labeled directly or indirectly with radiolabeled building blocks. Accessorily, cross-coupling reactions can be applied for indirect labeling due to the mostly mild reaction conditions and the short reaction times which are of major importance in radiochemistry. Therefore, it is possible to use these reactions in rapid and facile labeling syntheses involving the introduction of short-lived nuclides carbon-11 and fluorine-18. Advantageous, these coupling reactions allow highly selective and functional group-tolerating syntheses of radiotracers bearing both radionuclides.

The present review summarizes recent developments in the field of labeling procedures using transition metal-mediated cross-coupling reactions for the introduction of the short-lived positron emitting radionuclides carbon-11 and fluorine-18 into organic molecules. Applications for radiolabeling using the Cu-mediated "click chemistry" have been reviewed in detail elsewhere [3,4] and will not be discussed here.

## 2. Carbon-11 Labeling

**Scheme 1:**
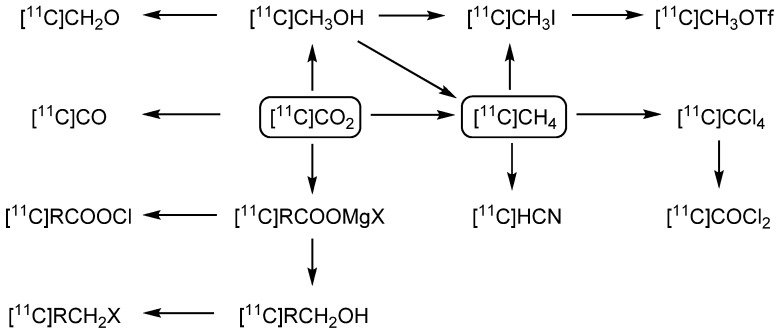
^11^C-labeled primary building blocks and resulting secondary ^11^C labeling units.

Carbon-11 is one of 13 known isotopes of the element carbon [5]. It is cyclotron-produced by a proton beam of about 10–17 MeV from a nitrogen-14 target which has previously been filled with a low concentration of oxygen or hydrogen. For this purpose, the ^14^N(p,α)^11^C nuclear reaction is exploited with a maximum theoretical specific activity of 3.4∙10^5^ GBq·µmol^−1^ [6,7,8] which is never reached in practice. Carbon-11 is a suitable nuclide for PET imaging because of its high positron emission rate (99.8%) and its low energy positrons (1.0 MeV). Another advantage of carbon-11 is based on its chemical equivalence to carbon-12 and carbon-13, when incorporated into bioactive compounds for PET imaging purposes. Incorporation of carbon-11 results in equivalent, bioactive radiopharmaceuticals. In contrast, in particular fluorine-18 usually replaces hydrogen atoms or hydroxyl groups and might therefore be responsible for a different (bio-)chemical behavior of the resulting radiotracer [7,8]. Due to its short half-life of 20.4 min, radiosyntheses utilizing carbon-11 are generally performed within 60 min including purification of the crude radiolabeled product [9]. This fact led to the development of rapid 'on-line'-syntheses, by which the primary building block can be converted into the radiochemical compound, after the carbon-11 species has been transferred from the cyclotron directly to the hot cell. Carbon-11 usually arrives in the chemical form of [^11^C]CO_2_ or [^11^C]CH_4_ from the cyclotron, which represent the two most important ^11^C-labeled primary building blocks [10] from which nearly all other ^11^C-labeling units are produced (Scheme 1).

**Scheme 2:**
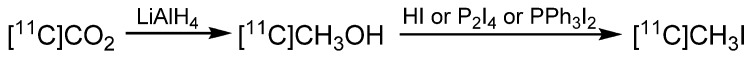
"Wet" chemistry route yielding [^11^C]methyl iodide.

Two main methods for the production of [^11^C]methyl iodide as the most important and most widely used secondary [^11^C]building block are stated in the literature. One approach, referred to as "wet" chemistry method, deals with the reduction of [^11^C]CO_2_ to [^11^C]methanol utilizing LiAlH_4_ in THF or diethyl ether. The resulting [^11^C]methanol is then converted into [^11^C]methyl iodide by the use of hydroiodic acid, diphosphorus tetraiodide [9] or triphenylphosphane diiodide [10], respectively (Scheme 2). Afterwards, the resulting [^11^C]methyl iodide is dried and separated from by-products.

**Scheme 3:**
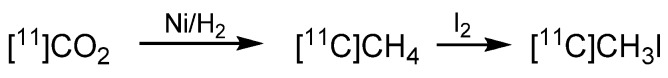
"Gas phase" chemistry route to [^11^C]methyl iodide.

The second approach, also known as "gas phase" method, deals with the conversion of [^11^C]methane into [^11^C]methyl iodide by radical iodination with elemental iodine at 700–750 °C (Scheme 3). Depending on the primary radiolabeled building block produced by the cyclotron, it might be necessary to convert [^11^C]CO_2_ into [^11^C]CH_4_ beforehand by the use of a nickel catalyst [11], otherwise [^11^C]CH_4_ is used directly [12].

The "gas phase" method offers several advantages. Due to the direct synthesis of [^11^C]methyl iodide, highest specific activities of max. 4,700 GBq·μmol^−1^ can be achieved [13], compared to 74–370 GBq·μmol^−1^ by the "wet" method [14]. The low specific activity obtained by the "wet" method is a result of contamination from naturally occurring [^12/13^C]CO_2_, which originates from LiAlH_4_ of the reduction step. Carbon-12/13 contamination may also arise from solvents and chemical impurities.

**Scheme 4:**
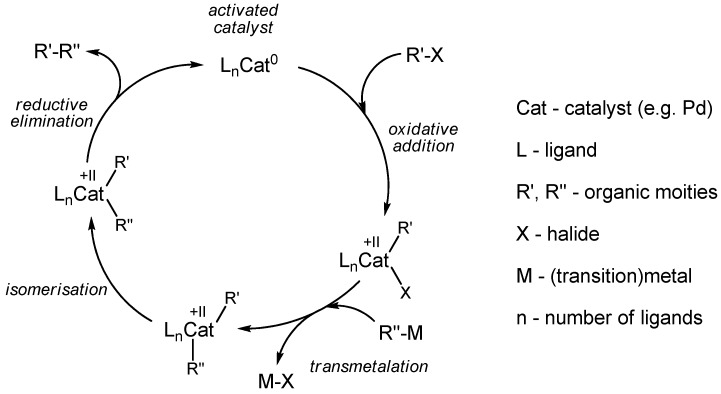
Proposed classical mechanism of cross-coupling.

The most prominent and most commonly applied procedure for the radiolabeling with carbon-11 represents the nucleophilic [^11^C]methylation of alcohols, primary and secondary amines, thiols, carbanions or carboxylic acid derivatives with [^11^C]methyl iodide (or [^11^C]methyl triflate). Besides, transition metal-mediated cross-coupling reactions are gaining in importance for labeling reactions with carbon-11. Although the mechanism of cross-coupling is not fully understood, most intermediates have been identified, supporting the proposed classical mechanism depicted in Scheme 4. In most cases, the catalyst is a palladium-0 species. According to this theory, the reaction steps proceed as follows: An organic halide R'-X is coordinated to the Pd-catalyst by oxidative addition. A second (metal)organo compound R''-M reacts with the activated Pd species by transmetalation in the rate-determining step. After isomerization, the desired cross-coupled compound R'-R'' is formed via reductive elimination in the final step. The catalyst is released in the process, is thereby regenerated and available for the next catalysis cycle.

### 2.1. Cross-coupling reactions with palladium

#### 2.1.1. Stille reaction

One of the mildest and most applied cross-coupling methods is the Stille reaction [15,16]. Organotin compounds function as starting material and alkyl/aryl halogenides as coupling partners, which are cross-coupled by the use of Pd-catalyst and a phosphane-based supporting ligand (PR_3_). A wide variety of functional groups such as amino, hydroxyl, thiol or carboxylate is tolerated. Another benefit of the Stille reaction is the stability of the organotin compounds against Brønsted acids and bases. However, one major drawback of organotin reagents used for the transmetalation step is their inherent toxicity in contrast to other starting materials like boronic acids. This may limit the application of this kind of cross-coupling reactions especially when pharmaceuticals are prepared.

**Scheme 5:**
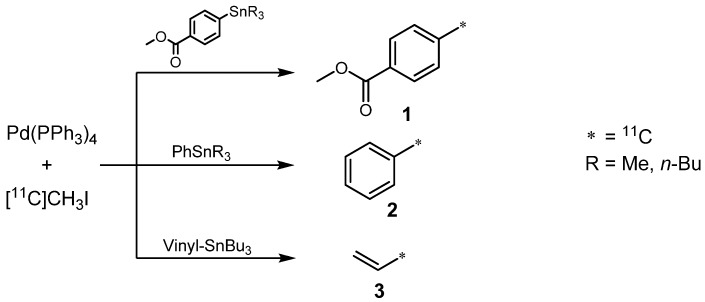
First approach of ^11^C-labeling by the Stille reaction.

The first attempt for the introduction of carbon-11 in molecules by the Stille reaction was reported in a work of Andersson *et al.* in 1995. Several organotin sample molecules were used for the synthesis of ^11^C-labeled model arenes **1** and **2**, as well as alkene **3** with radiochemical yields (RCYs) from 30 to 85% (Scheme 5) [17]. Optimizations to increase RCY and radiochemical purity were performed by exchanging solvents and stannane residues. Best results were found for aromatic trimethylstannyl compounds in DMF or DMSO. It was thereby demonstrated that the Stille reaction is a versatile tool for syntheses of ^11^C-labeled PET tracers as seen in Scheme 5.

An attempt for a general radiolabeling strategy with carbon-11 using the Stille reaction was published years later [18]. Therefore, high numbers of aromatic model stannanes were labeled with [^11^C]methyl iodide under different conditions. Best results were obtained when Pd_2_(dba)_3_ was used with P(*o*-Tol)_3_ as co-ligand and CuCl/K_2_CO_3_ as additive in DMF. 

**Scheme 6:**
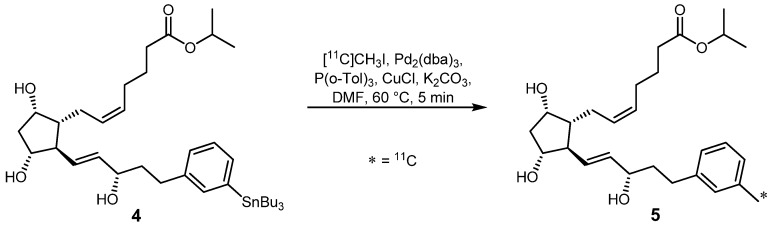
Synthesis of 17-(3-[^11^C]methylphenyl)-18,19,20-trinor-PGF_2α_ isopropyl ester (**5**).

Based on these investigations, Björkman *et al.* presented the preparation of stannylated prostaglandin analogue **4** which was used as precursor for a ^11^C-labeling in an improved Stille reaction (Scheme 6).

The resulting radiotracer **5** is based on the known PGF_2α_ drug Xatalan^®^ and is applied for studies of prostaglandin (PG) receptors in the brain. Compound **5** was obtained in 34% RCY decay-corrected (d.c.) within 30 minutes after end of bombardment (EOB) with a specific activity of 100 GBq·µmol^−1^ and a radiochemical purity >95% [19].

**Scheme 7:**
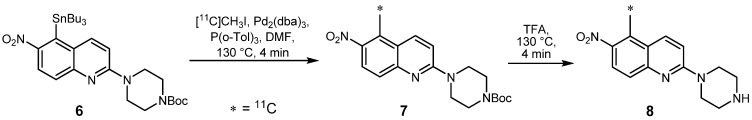
Synthesis of 5-HTT inhibitor [^11^C]5-methyl-6-nitroquipazine (**8**).

In 2002, Sandell *et al.* used similar conditions (Pd_2_(dba)_3_/P(*o*-Tol)_3_, DMF) for the preparation of [^11^C]5-methyl-6-nitroquipazine (**8**), an inhibitor for the serotonin (5-hydroxytryptamine) transporter (5-HTT) [20]. In contrast to the reaction conditions reported before [18,19], neither CuCl nor K_2_CO_3_ were added. In consequence of this omission, a higher reaction temperature (130 °C) and longer reaction time (~40 min after EOB) were required. However, compared to synthesis of **5**, these reaction conditions delivered higher RCYs (60–80%) and radiochemical purities (>99%) of **8**, but with a specific activity of only 19 GBq·µmol^−1^ after the two step synthesis depicted in Scheme 7. Upon radiosynthesis, compound **8** was tested as PET-tracer for the serotonin transporter 5-HTT in monkey brains. Unfortunately, it exhibited brain uptake kinetics which were too slow to justify further investigations.

**Scheme 8:**
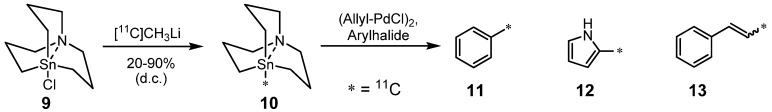
Examples of ^11^C-labeled products synthesized from **10**.

Due to the presence of other functional groups in the precursor like alcohols or amines, side reactions may occur [21]. Furthermore, preparation and purification of highly functionalized stannyl compounds can be difficult at times. In order to prevent such obstacles during the labeling process with [^11^C]methyl iodide and functionalized stannyl compounds, Forngren *et al.* presented the synthesis of the novel organotin compound **10**. This molecule already contains the [^11^C]methyl group and is well suited for radiolabeling purposes under Stille conditions. In this case, various organohalides were labeled using the novel ^11^C-containing stannane **10** [22]. First, [^11^C]methyl iodide was converted into [^11^C]methyl lithium [23] and subsequently mixed with 5-chloro-1-aza-5-stanna-bicyclo-[3.3.3]-undecane (**9**) in order to form 5-[^11^C]methyl-1-aza-5-stannabicyclo[3.3.3]undecane (**10**) with RCYs ranging from 20 to 90% (d.c.) related to [^11^C]methyl iodide (Scheme 8). As stated by the authors, the wide range in yields observed when preparing **10** might to some extent be explained by the presence of proton sources in the reaction mixture, which results in the formation of [^11^C]methane as a side-product.

The following cross-coupling reaction was arranged successfully in a 'one pot' procedure in which various functionalized organohalides were tested with regard to their applicability as precursor. Depending on reaction temperature and time as well as the employed arylhalide, maximum RCYs of 9% for **11**, 47% for **12**, and 90% for **13** were achieved with respect to [^11^C]stannane **10**. Unfortunately, no labeling products and only moderate yields were obtained at ambient temperature and at 50–65 °C, respectively. Best results were achieved at 100 °C in DMF with allylpalladium chloride dimer as mediator, reaction conditions which starkly limit the number of potential biomolecule precursors.

**Scheme 9:**
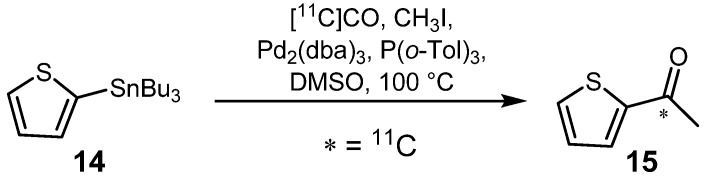
Synthesis of **15** by the Stille reaction.

Automation of radiosyntheses is always desirable, which is also the case for the Stille reaction. In 2005, Karimi *et al.* demonstrated a straightforward procedure at which [^11^C]CO was used in the palladium-mediated carbonylative coupling of organic iodides with organostannanes to synthesize various unsymmetrical alkyl and aryl-[^11^C-*carbonyl*]ketones [24]. According to the authors, this method could be automated easily, although no further application has been reported to date. The syntheses carried out were fast and led to 20 different ^11^C-labeled molecules with RCYs ranging from 37 to 98% and specific activities of up to 300 GBq·µmol^−1^. Different reaction conditions were applied in order to scrutinize scope and limitations of the respective Pd-catalyst, the co-ligands, the temperature and the solvent. Highest RCYs were obtained in DMSO at 100 °C with an excess of P(*o*-Tol)_3_ relative to Pd-catalyst, which is depicted exemplarily for radiosynthesis of compound **15** (Scheme 9).

**Scheme 10:**
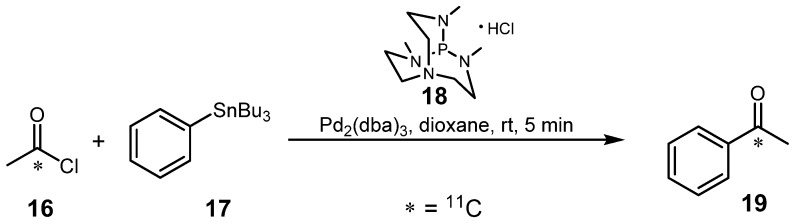
Improvement of Stille reaction using **16** as starting material and **18** as co-ligand.

Arai *et al.* improved the method established by Karimi *et al.* [24] (*vide supra*) which was applied as previously described by using a different co-ligand for the Pd-catalyst and [1-^11^C]acetyl chloride (**16**) instead of [^11^C]CO as depicted in Scheme 10 [25].

Co-ligands PPh_3_ and P(*o*-Tol)_3_ did not support synthesis of the desired model substance [*carbonyl*-^11^C]acetophenone (**19**). In contrast, 2,8,9-trimethyl-2,5,8,9-tetraaza-1-phosphabicyclo[3.3.3]undecane hydrochloride (**18**) showed a significant improvement of the yield when used in a ratio of 1:0.5 (co-ligand **18** to Pd_2_(dba)_3_). Finally, it was possible to obtain similar RCYs (60%; d.c.), yet under milder reaction conditions (ambient temperature) than reported by Karimi *et al.* for the preparation of compound **19**. The authors mentioned that chloride in the co-catalyst is critical in the mechanism, which still remains unclear, than the support of co-catalyst **18** without HCl. Furthermore, utilization of triaminophosphane hydrochloride **18** is advantageous in the regard that this compound is easy to handle and is stable against moisture and air [26].

**Scheme 11:**
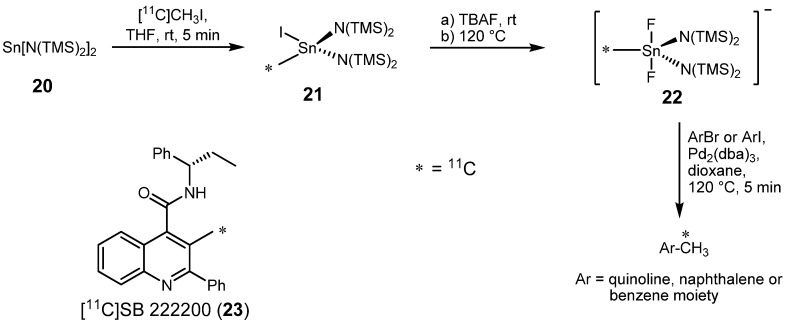
Stille reaction with monomethyltin reagent **22**.

In 2006, Huiban *et al.* reported further improvements of the Stille reaction for radiochemical purposes. An application of less toxic mono-alkylstannanes instead of previously used di-, tri-, or tetra-alkylstannanes delivered higher radiochemical yields and allowed a facile removal of inorganic byproducts [27]. In a first step, [^11^C]methyl iodide was reacted with Lappert’s stannylene Sn[N(TMS)_2_]_2_ (**20**) [28] quantitatively (Scheme 11). Obtained mono[^11^C]methyl tin compound **21** was activated thereafter by addition of tetra-*n*-butylammonium fluoride at ambient temperature leading to stannate **22**. The subsequent Pd-mediated Stille reaction of **22** with aryl bromides and iodides led to a wide variety of desired labeled heteroarenes with high RCYs (63 to 78%). Since no co-ligand like PPh_3_ was used, a formation of by-products (biaryl compounds), often formed by an aryl exchange reaction of such co-ligands with aryl substrates, was prevented. Based on this method, a new radiotracer [^11^C]SB 222200 (**23**) for the non-invasive study of functions and diseases involving cerebral neurokinin and opiate receptors, such as anxiety, depression, psychosis, schizophrenia and Parkinson’s disease [29], was prepared by the same working group [30]. However, no biological application has been reported to date.

Selected target molecules have been radiolabeled with carbon-11 via standard Stille reaction before, thus enabling a comparison of these methods. The results of this study confirmed the group’s previous findings, as similar or higher RCYs and specific activities, respectively, were obtained by using the improved reaction conditions. Thus, the authors postulate that their method, whenever applicable, is superior due to easier purification of ^11^C-labeled products, less toxicity of the used organotin compounds along with similar RCYs. A wide range of ^11^C-labeled radiotracers has been developed using conditions similar to the typical Stille reaction.

**Figure 1:**
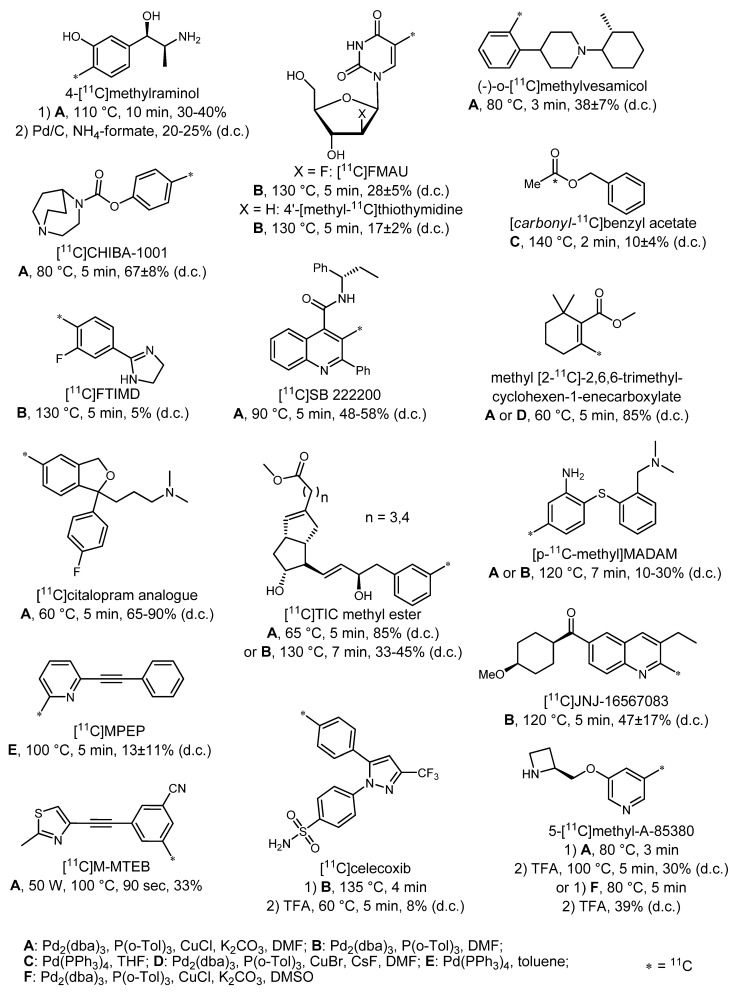
Overview of [^11^C]radiotracers prepared by Stille reaction.

Figure 1 shows an overwiev, including 4-[^11^C]methylraminol, a tracer to assess myocardial sympathetic innervation [31] as well as nucleosides such as [^11^C]FMAU [32] and 4'-[*methyl*-^11^C]thiothymidine [33] which are adopted for cell proliferation imaging.

With (–)-*o*-[^11^C]methylvesamicol [34] and ([^11^C]CHIBA-1001) [35], tracers for vesicular acetylcholine transporters as well as *α*7 nicotinic receptors, respectively, were developed successfully. Furthermore, a tracer for imaging of glial metabolism ([*carbonyl*-^11^C]benzyl acetate) was prepared [36] as well as [2-^11^C]-2,6,6-trimethylcyclohexen-1-eneyl carboxylic acid methyl ester, a potential ligand for the nicotinic acetylcholine receptor (nAChR) [37], a ligand for I_2_-imidazoline receptors ([^11^C]FTIMD) [38], and [^11^C]SB 222200, an antagonist of the neurokinin-3 receptor [39]. In addition, tracers for the serotonin transporter like the [^11^C]citalopram analog [40] and [(*p*-^11^C)methyl]MADAM [41] as well as ligands for metabotropic glutamate subtype 5 receptor, [^11^C]MPEP [42] and [^11^C]M-MTEB [43], respectively, were prepared. Another example is a radiocarbonylated ligand for the glutamate 1 receptor, [^11^C]JNJ-16567083 [44]. The prostaglandins [^11^C]TIC methyl ester [45,46,47], cyclooxygenase-2 inhibitor [^11^C]celecoxib [48] and nicotinic acetylcholine receptor ligand 5-[^11^C]methyl-A-85380 [49,50] were also found in the literature.

#### 2.1.2. Suzuki reaction

Cross-coupling reactions under Suzuki conditions with mostly nontoxic organoboron derivatives as transmetalation reagents are largely unaffected by the presence of water and tolerate a wide variety of functionalities [51,52,53]. The main advantage of this method is the ability for the cross-coupling of sp^3^-sp^3^ hybridized carbon species in contrast to other cross-coupling methods. Therefore, a wide range of alkyl, 1-alkenyl and arylboron reagents can undergo the palladium-catalyzed coupling reactions with alkyl, allyl, 1-alkenyl, 1-alkynyl and aryl substrates.

**Scheme 12:**
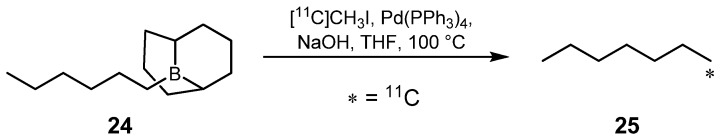
First radiolabeling with [^11^C]methyl iodide using the Suzuki cross-coupling.

A method for the rapid incorporation of carbon-11 was presented by Andersson and co-workers in 1995 using the Suzuki cross-coupling [17]. For this purpose, 9-hexyl-9-BBN (**24**) was converted into 1-[^11^C]heptane (**25**) in a sample reaction with a RCY of 35% and a radiochemical purity >95% (Scheme 12). The labeling reaction was carried out using [^11^C]methyl iodide and Pd(PPh_3_)_4_ as catalyst with THF as solvent under basic conditions.

**Scheme 13:**
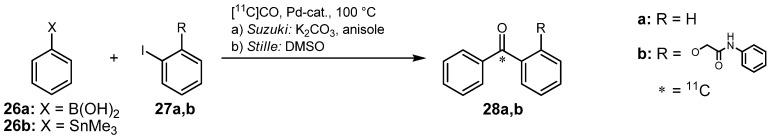
Suzuki and Stille reaction with [^11^C]CO.

A novel approach for the preparation of no-carrier-added (n.c.a.) [^11^C]CO from [^11^C]CO_2_ was presented in 1997 using elemental molybdenum instead of typically employed zinc metal [7]. A first attempt to use [^11^C]CO in Suzuki cross-coupling reactions was made [54], and [^11^C]benzophenone (**28a**) was chosen as target compound (Scheme 13). For this purpose, phenylboronic acid (**26a**) and iodobenzene (**27a**) were converted into **28a** in a Pd-mediated reaction using Pd(PPh_3_)_2_Cl_2_ as catalyst, K_2_CO_3_ as base and DMSO as solvent at 100 °C. After 16 min synthesis time, the desired product was obtained in 69% RCY (d.c.) with >99% radiochemical purity and a specific activity of 33 GBq·μmol^−1^.

Another example of the use of the Suzuki reaction in comparison to the Stille reaction for labeling purposes with carbon-11 was presented in 2002 [55]. The structurally more complex, unsymmetrical benzophenone [*carbonyl*-^11^C]2-(2-benzoylphenoxy)-*N*-phenylacetamide (**28b**) was developed which functions as inhibitor of the reverse transcriptase of the human immunodeficiency virus type 1 (HIV 1), (Scheme 13). For this purpose, trapped [^11^C]CO gas was released into a vessel containing phenylboronic acid (**26a**), Pd(PPh_3_)_2_Cl_2_, K_2_CO_3_ in anisole and 2-iodophenylacetamide (**27b**). K_2_CO_3_ is used for the elimination of the dihydroxyboron residue in the transmetalation step. Unfavorably, K_2_CO_3_, which is insoluble in anisole, led to the adsorption of the carbon-11-labeled product **28b** on the solid surface of K_2_CO_3_ and hence to a RCY of 20% only. Therefore, Stille reaction conditions were scrutinized by the authors as well, and yields and scope were compared to Suzuki conditions [55]. For this purpose, trimethyl(phenyl)stannane (**26b**) and **27b** were reacted with Pd(PPh_3_)_4_ in DMSO under Stille conditions. As stated above, inhibitor **28b** was obtained in 20% RCY in case of Suzuki conditions, whereas 71% RCY was achieved when Stille reaction conditions were applied. Obtained radiochemical purities >99% and specific activities >30 GBq·µmol^−1^ were similar with both synthesis routes. In this case, the Stille reaction is superior to the Suzuki reaction.

**Scheme 14:**
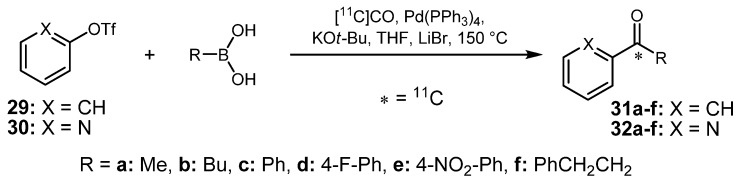
Examples of the improved Suzuki reaction.

In 2004, Rahman and co-workers applied [^11^C]CO for a labeling technique allowing the synthesis of [^11^C-*carbonyl*]compounds in high radiochemical yields and with good specific activities in relatively short synthesis times [56]. Due to the low reactivity and solubility of [^11^C]CO in organic solvents, labeling reactions were carried out in a fully automated system [57]. Thus, the cyclotron-produced [^11^C]carbon monoxide was concentrated to a small volume, trapped on liquid nitrogen-cooled silica and transferred into a micro-autoclave that could be pressurized to 40 MPa. Different functionalized aromatic boronic acid derivatives and boronic esters were tested and a variation of applied additives and bases led to an increased RCY for ^11^C-labeled compounds (Scheme 14). Regarding the starting materials, it was pointed out in a previous publication that the use of aryl triflates represents a good alternative to aryl iodides and delivers higher RCYs of the respective labeled products [58]. A further improvement was achieved by the addition of bases such as *tetra*-butylammonium fluoride or potassium *tert*-butoxide which increases the RCY in most cases. These bases are believed to facilitate the formation of a boronate, which in turn supports the transmetalation step with palladium. Furthermore, lithium bromide was added to prevent catalyst decomposition or to promote the cross-coupling carbonylation reaction. The best results were obtained in a sample synthesis, in which pyridin-2-yl triflate (**30**) was reacted with phenylboronic acid (**26a**) and potassium *tert*-butoxide as base, leading to the formation of [*carbonyl-*^11^C]ketone **32c**. The RCY was higher with KO*t*-Bu (78%) than without the use of a base (59%). It was discovered that in general, the RCY highly depends on the use of the respective boronic acid and the base: *tetra*-butylammonium fluoride delivered higher RCYs when used for cross-coupling purposes with alkyl boronic acids, whereas potassium *tert*-butoxide was more suitable when aromatic boronic acids were applied.

**Scheme 15:**
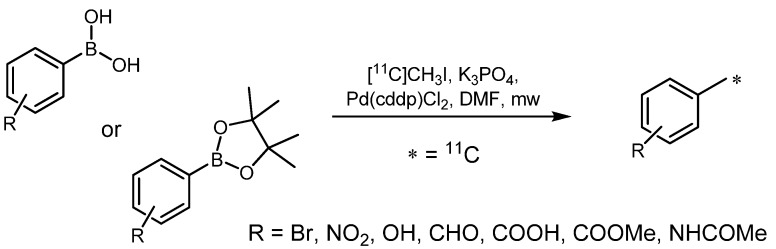
Suzuki reaction with different organoboron derivatives.

A further improvement of the Suzuki reaction for the preparation of carbon-11-labeled toluene derivatives was reported by Hostetler *et al.* in 2005. They found that the addition sequence of starting materials is crucial for the preparation of radiolabeled compounds in high RCYs [59]. The oxidative addition to the palladium complex has to be executed first when using trace amounts of alkyl halides, as it is the case in radiosyntheses. Therefore, first of all [^11^C]methyl iodide was distilled directly into the reaction vessel containing Pd(dppf)Cl_2_ dissolved in DMF (Scheme 15). Secondly, organoboron compound and base were added. Although the palladium catalyst is hardly soluble in DMF, this combination resulted in the highest RCYs, whereas the addition of typically used Pd(PPh_3_)_4_ catalyst led to palladium black as by-product along with lower RCYs. Another improvement was achieved by varying the used base. Aqueous KOAc and aqueous Cs_2_CO_3_ resulted in no product formation, whereas aqueous NaOH led to increased RCYs. However, aqueous K_3_PO_4_ was the mildest base and yielded the desired product in high RCYs. In general, a short treatment with microwaves (90 s) still raised the RCYs. In addition, application of different organoboron species was scrutinized, however, all investigated boron species showed similar RCYs. Acidic proton possessing moieties like 4-hydroxyphenylboronic acid pinacol ester as well as 4-carboxybenzeneboronic acid pinacol ester could be used in combination with the base K_3_PO_4_ without any difficulty. In these reactions a maximum RCY of 92% (d.c.) was reached for the preparation of *p*-[^11^C]cresol with >95% radiochemical purity in less than 20 minutes according to the release of [^11^C]methyl iodide.

After the Suzuki reaction was proven to be a versatile and mild cross-coupling method for the introduction of [^11^C]CO or [^11^C]methyl iodide with good RCYs, an application for the preparation of ^11^C-labeled PET-tracers using cross-coupling seems to be a reasonable approach. However, no further applications for this synthesis strategy were found in the literature to date.

#### 2.1.3. Heck reaction

**Scheme 16:**
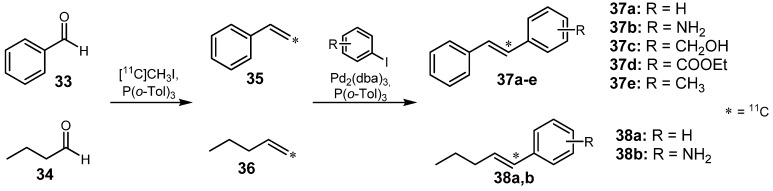
Heck reaction for [^11^C]-labeled olefins **37a-e** and **38a,b**.

The Heck reaction does not follow the classical mechanism of a cross-coupling reaction, but also involves a palladium-catalyzed C-C-bond formation between olefins and aryl/vinyl halides [60]. Björkmann and Långström described a method in which ^11^C-labeled olefins **35** and **36** were prepared and subsequently cross-coupled using a Pd-mediated Heck reaction yielding (*E*)-[^11^C]stilbenes **37a-e** and (*E*)-[1-^11^C]pent-1-enes **38a,b**, respectively (Scheme 16) [61]. During this reaction sequence, [^11^C]methyl iodide was incorporated in a first step into benzaldehyde (**33**) and butyraldehyde (**34**) via Wittig olefination to form [^11^C]styrene (**35**) and [1-^11^C]pent-1-ene (**36**), respectively. Since traces of triphenylphosphane as co-ligand were able to perform an aryl exchange in the Pd-complex with the appropriate substituted aryl halide, [^11^C]stilbene was formed as by-product in all preparations. To circumvent this side reaction, the use of tri-*o*-tolylphosphane in the primary Wittig olefination as well as in the subsequent Heck reaction was essential to obtain the desired coupling products. Additionally, an excess of Pd species was necessary in the Heck reaction in order to transform the [^11^C]olefins **35** and **36** quantitatively. Of the model compounds depicted in Scheme 16, highest RCYs were obtained for **37b** (40%) and **38b** (54%) (d.c.) under optimized conditions (150 °C, 5 min). In both cases, the radiochemical purity achieved was greater than 95% after 40 min total synthesis time.

#### 2.1.4. Sonogashira reaction

Like the Heck reaction, the Sonogashira reaction belongs to cross-coupling reactions although it follows a different mechanism compared to classical cross-coupling methods like Suzuki or Stille. In case of the Sonogashira reaction, the difference to classical cross-coupling mechanisms is the formation of an intermediate organocopper species which interacts with the Pd-catalyst in a transmetalation step. Terminal alkynes, which are coupled with aromatic or vinylic halides, represent the principal ligands of the organocopper species [62].

**Scheme 17:**
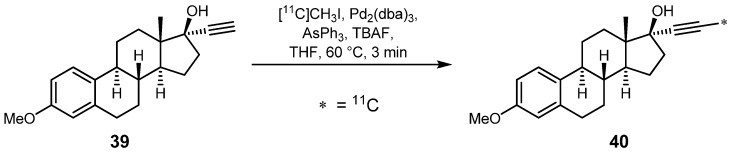
Sonogashira-like reaction with terminal alkynyl steroid **39**.

Regarding radiolabeling with carbon-11, the only work found in the literature dealing with the Sonogashira reaction was published in 2003. Wuest *et al.* reported the radiolabeling of model steroid 17*α*-(3'-[^11^C]prop-1-yn-1-yl)-3-methoxy-3,17*ß*-estradiol (**40**) by means of [^11^C]methyl iodide (Scheme 17) [63]. Classical Sonogashira reaction conditions with copper(I) salts and bases such as triethylamine were tested but resulted in very low RCYs (1-6%) due to the consumption of [^11^C]methyl iodide by the used amine base. However, the reaction conditions were modified which finally led to preparation of ^11^C-labeled steroid **40** at RCYs in the range of 49 to 64% (d.c.). Terminal alkyne **39** was used as precursor as well as Pd_2_(dba)_3_, AsPh_3_ and tetra-*n*-butyl-ammonium fluoride. After a total synthesis time of 21-27 min, a radiochemical purity >99% was achieved, unfavorably along with quite low specific activities (10-19 GBq·µmol^−1^). Functional groups like the hydroxyl group were not affected and made this procedure a valuable tool for ^11^C-radiolabeling. 

#### 2.1.5. Miscellaneous cross-coupling reactions

**Scheme 18:**
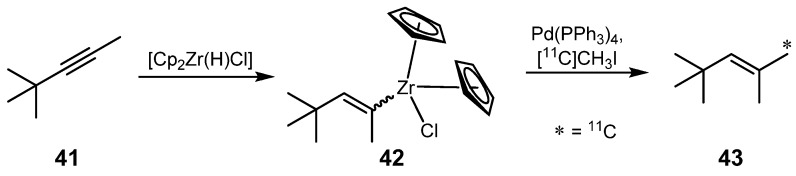
Synthesis of model compound 2,4,4-[^11^C]trimethyl-pent-2-ene (**43**).

In 2006, Wuest and Berndt described a novel palladium-mediated cross-coupling strategy for ^11^C-C bond formations utilizing alkenylzirconocene complexes [64], which represent a valuable tool for the preparation of ^11^C-labeled compounds containing a prenyl group. The synthesis route of model compound 2,4,4-[^11^C]trimethyl-pent-2-ene (**43**) is depicted in Scheme 18. (4,4-Dimethylpent-2-en-2-yl)zirconocene complex (**42**) was applied as precursor in this reaction and was obtained by *syn*-addition of the Zr-H bond of Schwartz reagent [Cp_2_Zr(H)Cl] onto an internal C-C triple bond of 4,4-dimethylpent-2-yne (**41**) [65]. Treatment of **41** with an excess (2.5 equivalents) of Schwartz reagent was necessary in order to form the thermodynamically stable alkenylzirconocene **42** exclusively. The authors demonstrated that this complex undergoes transmetalation with metal complexes M(PPh_3_)_4_ (M = Pd, Pt, Ni), at which this reaction step with Pd is superior to other investigated transition metals such as Pt or Ni. A subsequent cross-coupling was initiated and, upon addition of [^11^C]alkyl halides and 5 mol% of Pd(PPh_3_)_4_, new ^11^C-C bonds were formed under retention of the double bond configuration yielding prenyl derivatives such as model compound **43**. These reactions require an internal alkyne in order to present a good electrophile for the insertion step. In contrast, slightly reducible functions like esters or nitro groups do not yield the desired product due to the formation of alkenylzirconocenes followed by hydrozirconization. Various internal alkynes were applied, but the highest RCY of 75% based upon unreacted [^11^C]methyl iodide was obtained for compound **43**.

**Scheme 19:**
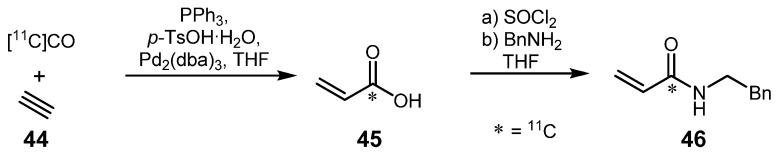
Preparation of [1-^11^C]acrylamide (**46**).

The acrylamide moiety is part of the structure of various bioactive compounds and could, after further chemical modifications, be used to design new PET tracers [66]. Previous methods for the synthesis of *N*-substituted [*carbonyl*-^11^C]acrylamides dealt with the use of [^11^C]carbon dioxide. In this case, [^11^C]CO_2_ is treated with the respective Grignard reagent to obtain the corresponding [1-^11^C]acrylic acid with a moderate specific radioactivity of 200–500 MBq·μmol^−1^ due to isotopic dilution originating from atmospheric carbon dioxide, which also reacted with the Grignard reagent [67]. To increase the specific radioactivity associated with an application of [^11^C]CO_2_, Eriksson *et al.* developed two novel procedures for the preparation of several [*carbonyl-*^11^C/^13^C]acrylamides [68]. The first method involved the palladium-catalyzed cross-coupling reaction between [^11^C/^13^C]CO and acetylene (**44**) to yield [1-^11^C]acrylic acid (**45**). This step was followed by chlorination with SOCl_2_ and subsequent treatment with benzylamine. The respective ^11^C-labeled acrylamide **46** was obtained in 51 ± 4% (d.c.) radiochemical yield based on [^11^C]carbon monoxide with a specific radioactivity of 330 ± 4 GBq·μmol^−1^ (Scheme 19). Co-labeling with [^13^C]carbon monoxide confirmed the labeling position via ^13^C-NMR spectroscopy.

**Scheme 20:**
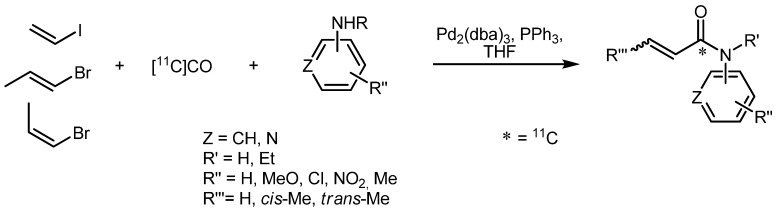
Examples of the 'one pot' synthesis yielding *N*-substituted [*carbonyl*-^11^C]acrylamides.

In the second approach by Eriksson *et al.*, several other *N*-substituted [*carbonyl*-^11^C]acrylamides were prepared from vinyl halides and amines in a novel 'one pot' synthesis procedure as depicted in Scheme 20. For this purpose, a Pd(0) complex was formed using Pd_2_(dba)_3_, PPh_3_ and *p*-toluenesulfonic acid monohydrate in THF [69]. The subsequent addition of vinyl halide caused a color change indicating the oxidative addition to the Pd complex. Afterwards, the amine was added and the solution was transferred to a micro autoclave containing [^11^C]CO, where the mixture was heated to 110 °C for 4 min. With this method, acrylamide **46** was obtained in significantly higher isolated RCYs (81 ± 3%) as compared to the first approach. Based on these improved results, several substituted amines were scrutinized for their reactivity with (*E*) and (*Z*)-isomers of 1-bromo-propene. RCYs ranged from 46 to 81% (d.c.) with radiochemical purities >97%. Of note, (*E*) and (*Z*)-isomers reacted with retention of their configuration. Compared to the method mentioned above using Grignard reagents, the specific radioactivity of *N*-benzyl[carbonyl-^11^C]acrylamide (**46**) was very high (330 ± 4 GBq·μmol^–1^).

**Scheme 21:**
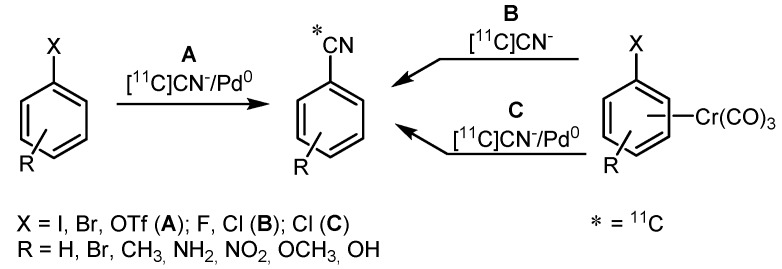
Pd/Cr-mediated cross-coupling with [^11^C]cyanide.

Since nitriles also comprise terminal triple bonds, the Pd-mediated cross-coupling with [^11^C]cyanide proceeds in a way similar to the Sonogashira reaction mentioned above. Andersson and Långström reported ^11^C-labeling of aryl halides and triflates by Pd-mediated [^11^C]cyanide cross-coupling [70]. This method included synthesis of several [^11^C]nitrile arenes and [^11^C]heteroarenes in a first approach, using aryl halogenides or aryl triflates as starting material and Pd(PPh_3_)_4_ as catalyst (Scheme 21, path **A**). Employment of iodide compounds resulted in the highest RCYs. The optimal solvent appeared to be THF (RCYs >99%) whereas acetonitrile or DMSO led to slightly lower RCYs (70–99%). In a second approach, the authors also investigated arene(tricarbony1)chromium complexes (Scheme 21, right). This approach (path **B**) entailed moderate to high RCYs (50–74%) in DMSO, which seemed to be the most suitable solvent under these conditions. Combination of both chromium and palladium-mediators (path **C**) resulted in a higher RCY (95%). Therefore, depending on the substrate, path **A** or **C** seem to be most suitable for this kind of reaction. 

**Scheme 22:**
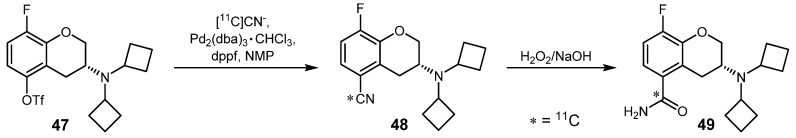
Synthesis of 5-HT_1A_ receptor antagonist [^11^C]NAD-299 (**49**).

In 1999, Sandell *et al.* reported about the potential 5-HT_1A_ receptor antagonist [^11^C]NAD-299 **49**, which was labeled with [^11^C]cyanide via a Pd-mediated cross-coupling [71]. It was mentioned that this PET tracer (K_i_ = 0.6 nM) should help finding serotonin transporter dysfunctions in the human brain. For that purpose, a straight forward method for the synthesis of compound **49** was developed. Reaction conditions evaluated by Andersson and Långström in 1995 [17] were applied and 1,1'-bis-(diphenyl-phosphino)ferrocene (dppf) was used as co-ligand and anhydrous *N*-methyl-2-pyrrolidinone (NMP) as assisting compound. Antagonist **49** was obtained in 20-40% RCY with a specific activity of 24 GBq·µmol^−1^ and a radiochemical purity >99% in a total synthesis time of 40-45 min. (Scheme 22) Biodistribution studies confirmed that [^11^C]NAD-299 (**49**) is a potential radiotracer for the 5-HT_1A_ receptor due to its high affinity and selectivity *in vivo*.

**Scheme 23:**
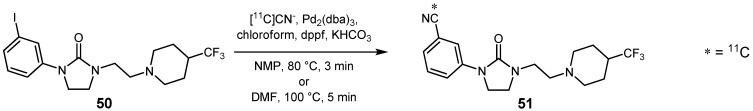
Synthesis of the PET tracer **51** for the dopamine D_3_ receptor.

Another approach for the synthesis of dopamine receptor radiotracers was published in 2009. Bennacef *et al.* used the [^11^C]cyanide cross-coupling for the synthesis of the dopamine D_3_ receptor antagonist **51**, which was applied as radiotracer for molecular imaging of Parkinson’s disease, schizophrenia or drug addiction [72]. A known potential structure (pK_i_ = 9.1) was used as template and a straightforward labeling method was framed (Scheme 23).

Antagonist **51** was prepared via a palladium(0)-catalyzed cross-coupling between precursor **50** and [^11^C]cyanide using Pd_2_(dba)_3_, CHCl_3_/dppf and KHCO_3_ as base in either NMP (80 °C, 3 min) or DMF (100 °C, 5 min). Afterwards, the crude product was purified by reverse-phase HPLC to yield 899 ± 326 MBq of **51** with a specific activity at end of synthesis (EOS) of 55.5 ± 25.9 GBq·μmol^−1^ and both chemical and radiochemical purities >99%. However, due to the fact that **51** has a too low pK_i_ and due to the low density of D_3_ receptors in brain, no specific signal was found using this radiotracer in animal PET studies.

### 2.2. Cross-coupling reactions with cuprates

**Scheme 24:**
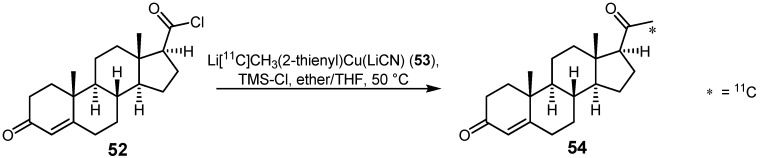
Synthesis of [21-^11^C]progesterone **54**.

In 1997, Lidström and co-workers used the novel developed lithium [^11^C]methyl(2-thienyl)cuprate (**53**) in order to produce [21-^11^C]-labeled progesterone **54** for its application in *in vitro* and *in vivo* PET studies [73]. The required cuprate **53** was synthesized via an exchange reaction of *n*-BuLi with [^11^C]methyl iodide, followed by the addition of lithium(2-thienyl)cyanocuprate. Deoxycorticosterone in the form of acid chloride **52** was reacted with [^11^C]cuprate **53** to yield [21-^11^C]progesterone **54** using cross-coupling conditions as depicted in Scheme 24. Synthesized carbon-11 labeled hormone **54** was obtained with a specific activity of 14 GBq·µmol^−1^ and a decay-corrected radiochemical yield of 30-35% within 35 min.

**Scheme 25:**
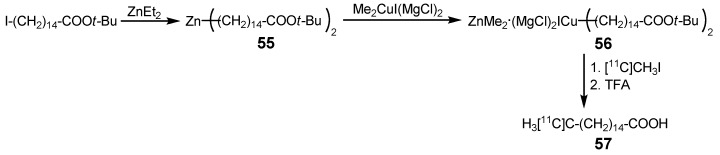
Synthesis of ^11^C-labeled palmitic acid **57**.

Amongst others, the fatty acid metabolism plays an important role as main energy source for the myocardium [74]. An accumulation of toxic metabolites in the mitochondria of these cells takes place when enzymatic dysfunctions interrupt the metabolism. Those toxic enrichments can cause sudden cardiac arrest. Therefore, fatty acids labeled with positron-emitting nuclides represent a promising approach to find toxic enrichments and thus probable cardiac issues. In 2000, Wuest *et al.* used the cross-coupling method developed by Tucker *et al.* [75] and produced the highly reactive dialkylzinc copper precursor **56** in order to synthesize radiolabeled fatty acids such as [^11^C]palmitic acid **57** with several [^11^C]alkyl iodides (Scheme 25) [76]. Cross-coupling reactions between functionalized dialkyl zinc compounds (ester, cyano groups, alkyl chains) and functionalized alkyl halides are highly chemoselective and result in good yields if certain cuprates (e.g. Me_2_Cu(CN)(MgCl)_2_) are used [75]. Interestingly, Wuest *et al.* recognized that the use of Me_2_CuI(MgCl)_2_ was essential for the reactivity of the copper-zinc species, when reacted with other halides. If Me_2_Cu(CN)(MgCl)_2_ or Me_2_CuI(MgBr)_2_ are used instead, only the cross-coupling with [^11^C]methyl iodide is successful. Furthermore, the authors of this study investigated different [^11^C]alkyl iodides (methyl, ethyl, *n*-butyl) as precursors and reacted them with the appropriate fatty acid moiety to obtain [ω, ω-1, ω-3-^11^C]palmitic acid, respectively. The utilization of [^11^C]methyl iodide as starting labeling material resulted in the highest decay-corrected RCY (16%) after 25-35 min in comparison to the other two [^11^C]alkyl halides (6% and 10%), most likely due to its higher reactivity.

### 2.3. Cross-coupling reactions with rhodium

**Scheme 26:**
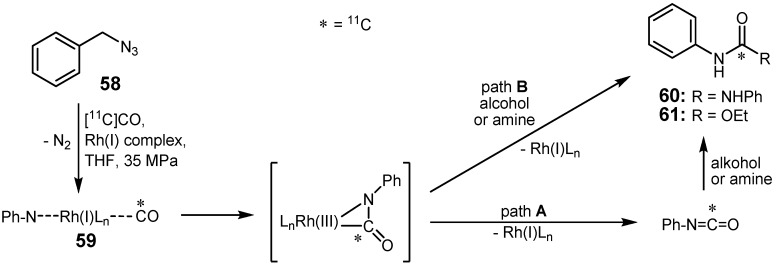
Presumed cross-coupling paths A and B for the formation of **60** and **61**.

The use of various Rh(I)-complexes was proven to be milder and less aggressive to thermosensitive molecules in contrast to other transition metal-catalyzed reactions [77]. Doi *et al.* used a rhodium-promoted carbonylation of phenyl azide (**58**) with [^11^C]CO and [^11^C]CN^-^ to synthesize *N*,*N'*-diphenyl[^11^C]urea (**60**) and ethyl phenyl[^11^C]carbamate (**61**), respectively [78]. The presumed reaction mechanism is shown in Scheme 26. The azide moiety binds to the rhodium complex, which has to be used in catalytic amounts of 0.01 molar related to phenyl azide, by abstracting nitrogen. Otherwise, the stoichiometric use of rhodium-complexes would involve the formation of a stable [^11^C]CO-coordinated Rh complex which would then not yield any ^11^C-labeled product. The authors pointed out that a strong nucleophile, such as a small amine, is needed to react with the [^11^C]isocyanate-coordinated Rh-complex **59**.

Carbon-11-labeled urea derivative **60** was obtained in 84% RCY, whereas a RCY of 70% was achieved for the formation of [^11^C]carbamate **61**, most likely due to the lower nucleophilicity of ethanol compared to aniline. The best catalytic system for [^11^C]CO trapping efficiency and product yield appeared to be [RhCl(cod)]_2_ (cod = 1,5-cyclooctadiene) with two equivalents of 1,2-bis-(diphenylphosphino)ethane (dppe) as co-ligand. 

**Scheme 27:**
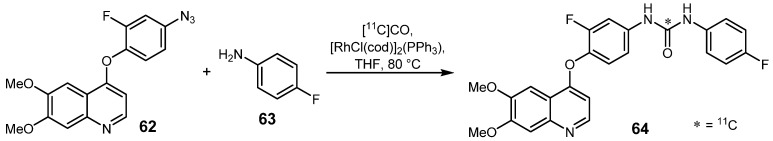
Rhodium-mediated cross-coupling of azide **62** with amine **63**.

Ilovich and co-workers adapted a method [79] which was originally elaborated by La Monica *et al.* [80]. They designed the ^11^C-labeled urea derivative **64** which is a promising VEGFR-2/PDGFR-*ß* inhibitor and was applied as PET tracer *in vivo* in order to image tumor-induced vascularization. The cross-coupling of azide **62** and amine **63** with [^11^C]CO was performed with a catalytic amount of [RhCl(cod)]_2_ with PPh_3_ or dppe as co-ligand (Scheme 27). Surprisingly, the use of PPh_3_ significantly decreased the required reaction temperature from 140 °C down to 80 °C and moreover increased the RCY to 81% in contrast to 44% RCY yielded with dppe as co-ligand. The authors postulated that azide **62** probably forms a nitrene-metal complex under cleavage of N_2_. In the next step, [^11^C]CO presumably reacts with the coordinated nitrene under formation of a ^11^C-N bond. The presence of an amine in the initial solution is essential for a one-step reaction, since the amine can react either directly with the metal-carbonyl complex or with the *in situ* produced isocyanate to obtain the urea. An employment of small amines like **63** is essential to achieve high radiochemical yields and to avoid side products due to less sterical hindrance and the amine's higher solubility, compared to other primary amines. Compound **64** was obtained in 81% RCY (d.c.) at a specific activity of 92 ± 4 GBq·µmol^−1^ 45 min after EOB.

## 3. Fluorine-18 Labeling

Fluorine-18, one of 17 known radioactive isotopes of fluorine [5], is currently the positron-emitting radioisotope of choice for the development of PET radiopharmaceuticals. The high interest for fluorine-18 applied in nuclear medicine is related to its favorable physical and nuclear properties: With a half-life of 109.8 min, a high positron decay ratio of 97% and a low positron energy (635 keV maximum; 2.3 mm range in matter), the application of this radionuclide is particularly advantageous in terms of resolution and dosimetry [81].

Fluorine-18 can be prepared in two chemical forms with different chemical behavior. On the one hand, [^18^F]F_2_ is provided in an extremely reactive electrophilic form from the nuclear reaction ^20^Ne(d,α)^18^F. Essentially, fluorine-19 has to be added as carrier gas to the target which decreases the specific activity of [^18^F]F_2_ (<750 MBq·μmol^−1^). On the other hand, the nuclear reaction ^18^O(p,n)^18^F is widely used for the preparation of large amounts of nucleophilic [^18^F]fluoride with high specific activities (up to 185 GBq·μmol^−1^). Highly enriched oxygen-18 water with an isotopic abundance of 99.9% is used as target and in this case addition of a fluorine-19 species is not necessary. Fluorine-18 is obtained as [^18^F]fluoride (in form of K[^18^F]F as a result of K_2_CO_3_ addition) in aqueous solution and is subsequently transformed into a more nucleophilic species by water elimination using a azeotropic co-distillation procedure with acetonitrile. Accessorily, a nitrogen-containing cryptand (in most cases Kryptofix K 222) is added in order to reinforce the nucleophilicity of 'naked' fluoride [82]. The drying stage can be avoided by using an ionic liquid as reactive fluorination medium [83]. Due to the higher specific activity and the better handling, most of the radiotracers were prepared using [^18^F]fluoride. 

Simple organic bioactive molecules or complex macromolecules of biological interest such as peptides, proteins or oligonucleotides have been labeled with fluorine-18 successfully [84]. A direct labeling proceeds by the displacement of good leaving groups with fluorine-18 at the appropriate precursor. Sensitive organic structures and macromolecules cannot be labeled directly due to the harsh reaction conditions during the introduction of radiofluorine. Therefore, different approaches have been attempted. Typically, a fluorine-18-containing building block is prepared primarily which is subsequently coupled to the rest of the molecule under mild reaction conditions. 

Examples for radiolabeling reactions with the most widely used PET radionuclide fluorine-18 using transition metal cross-couplings were rarely found in the literature [85]. A SciFinder-based research (June 2010) lists only eleven examples compared to numerous examples of carbon-11 radiotracers prepared using Pd-mediated cross-coupling reactions. As the 4-fluorophenyl group is frequently encountered in molecules of biological or medicinal interest, most radiofluorination procedures comprise the introduction of ^18^F-labeled aryl halides using carbon-carbon cross-couplings like Suzuki, Stille or Sonogashira. These methods can be regarded as mild and efficient procedures for the insertion of a 4-[^18^F]fluorophenyl moiety into various small organic molecules. Alongside, labeling procedures under Buchwald-Hartwig conditions as well as under conditions of the Ullmann type condensation led to radiofluorinations via carbon-heteroatom cross-coupling.

### 3.1. ^18^F-labeled compounds prepared via carbon-carbon cross-coupling reactions

**Scheme 28:**
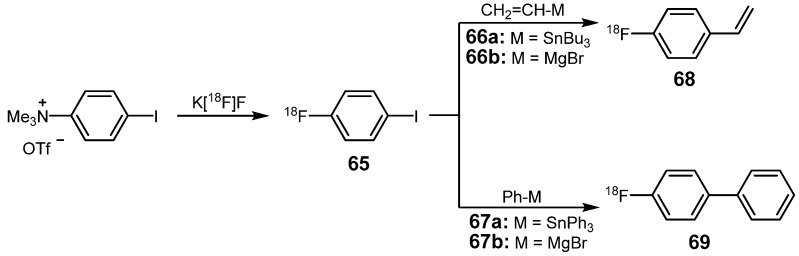
Fluorine-18-labeled styrene **68** and biphenyl derivative **69**.

The 4-fluorophenyl group as the most prominent structural motif is frequently found in biologically active molecules and fluorinated drugs [86,87]. Thus, syntheses based on transition metal catalysis or mediation for the incorporation of this functional group were explored in the past. First examples were described in 1995 when the syntheses of two model compounds 4-[^18^F]fluorophenylethene (**68**) and 4-[^18^F]fluorobiphenyl (**69**) were reported (Scheme 28) [88,89].

Two synthesis routes were presented for the evaluation of this metal-mediated approach. The first procedure included the Pd-mediated cross-coupling of organotin compounds **66a** and **67a** (Stille cross-coupling), whereas the second approach exploited the application of Grignard-reagents **66b** and **67b** (Kumada cross-coupling). In both cases, fluorine-18 was introduced by the use of 1-[^18^F]fluoro-4-iodobenzene (**65**) which was prepared from the appropriate trimethylammonium precursor with [^18^F]fluoride via nucleophilic aromatic substitution. The trimethylammonium precursor was obtained in high yield via methylation of 4-iodo-*N*,*N*-dimethylaniline using methyl triflate [90]. 

Optimization of the conditions for the radiolabeling reaction of **65** with **67a** revealed that the highest RCY (86%) of **69** can be obtained when hexamethylphosphoramide is used as solvent and Pd(PPh_3_)_4_ as catalyst. In general, application of organostannanes **66a** and **67a** delivered higher yields (15–80%) compared to the respective Grignard reagents **66b** and **67b** (20–53%).

**Scheme 29:**
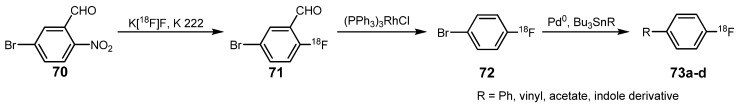
Syntheses of fluorine-18-labeled model compounds **73a-d**.

Fluvastatin is a prominent example of a bioactive compound containing the 4-fluorophenyl structural element. For this reason, a labeling method was investigated using palladium-promoted cross-coupling reactions of organotin compounds and aryl halides (Stille cross-coupling) for the introduction of fluorine-18 [91]. 5-Bromo-2-[^18^F]fluorobenzaldehyde (**71**) was prepared from the respective nitro-precursor **70** followed by subsequent decarbonylation with Wilkinson's catalyst [92] which afforded the actual labeling agent 1-bromo-4-[^18^F]fluorobenzene (**72**) in approx. 70% RCY (Scheme 29). In order to simplify the synthesis route, a 'one pot' procedure for the preparation of **72** was developed. Optimal reaction conditions [93] were elaborated for a subsequent radiolabeling of organostannanes with **72**. Best radiochemical yields were obtained utilizing Pd_2_dba_3_/AsPh_3_ as mediator in a DMF:dioxane mixture (1:1) at 115–120 °C. It was shown that the purification of **72** is important and increased the RCY of the coupling product from 20% to 90% with respect to the preparation of 4-[^18^F]fluorobiphenyl (**73a**). In order to label fluvastatin, 1-isopropyl-2-methylindolyl(tributyl)tin was prepared as synthon for the introduction of the 4-[^18^F]fluorophenyl group into the indole moiety (Table 1), however, no preparation of fluorine-18-labeled fluvastatin has been reported to date.

**Table 1:** Selected conditions and RCYs of the Pd-mediated cross-coupling with organostannanes.

Reagent	T / °C	t / min	Product	RCY / %
Bu_3_SnPh	115	15	4-[^18^F]fluorobiphenyl **73a**	>90
Bu_3_SnCH=CH_2_	115	30	4-[^18^F]fluorostyrole **73b**	>80
Bu_3_SnOOCCH_3_	120	5	4-[^18^F]fluorophenyl acetate **73c**	78
1-isopropyl-2-methyl-3-(tributylstannyl)indole	115	15	1-isopropyl-2-methyl-3-(4-[^18^F]fluorophenyl)indole **73d**	15

**Scheme 30:**
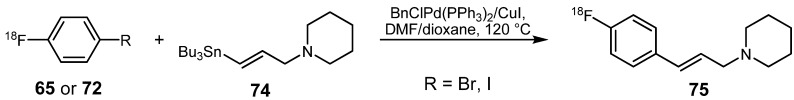
Preparation of model compound **75** utilizing **65** or **72**.

For the preparation of model compound **75**, functionalized stannane **74** was reacted with 1-[^18^F]fluoro-4-iodobenzene **65** or 1-bromo-4-[^18^F]fluorobenzene **72** as labeling agents using the Pd-mediated Stille cross-coupling [89]. The best reaction conditions were found to be a DMF:dioxane mixture (1:1) as solvent and BnClPd(PPh_3_)_2_:CuI (ratio 1:1) as catalyst at 120 °C. The regio and stereoselectivity of the coupling reaction were also investigated. It was observed that only the *E*-isomer of 4-[^18^F]fluorophenylallylpiperidine (**75**) was obtained (Scheme 30) when **65** or **72** were employed as starting material. A RCY of 90% was achieved when **65** was used compared to only 41% when **72** was used.

**Scheme 31:**
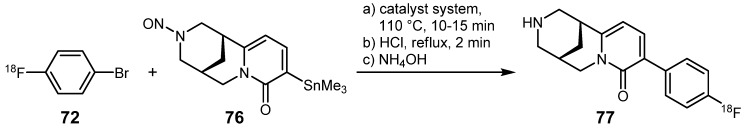
Preparation of radiofluorinated cytisine analog **77**.

Another application based on the previous work was published in 2000 for the synthesis of a cytisine analog [94]. This compound was prepared for *in vivo* studies of the *α*_4_*β*_2_ nicotinic acetylcholine receptor [95] using PET. The incorporation of fluorine-18 was put into practice by exploitation of the rapid and efficient Stille cross-coupling. As depicted in Scheme 31, 1-bromo-4-[^18^F]fluorobenzene (**72**) was prepared and reacted with 9-trimethylstannylcytisine **76**. Radiotracer **77** was received after a subsequent denitrosation. The highest radiochemical yields of the radiofluorinated cytisine derivative **77** were obtained using either a DMF/dioxane mixture and Pd(PPh_3_)_4_ (68%) or dioxane solely together with PdCl(PPh_3_)_2_ (56–74%) after 150 min total synthesis time starting from K[^18^F]F. The results were in good agreement with those obtained using the respective fluorine-19 compound but within a much shorter reaction time.

**Scheme 32:**
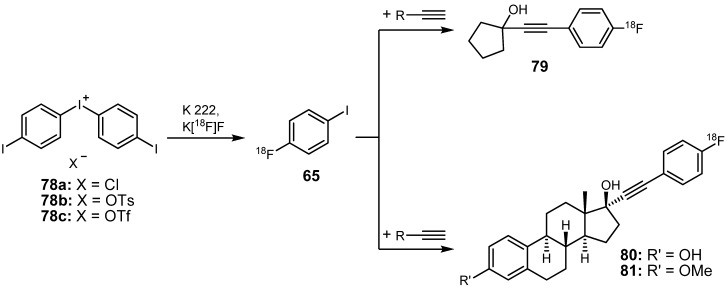
Preparation of **65** from iodonium salts and subsequent radiolabeling to obtain **79** as well as steroids **80** and **81**.

In 2003, Wuest *et al.* demonstrated the applicability of 4-[^18^F]fluoro-1-iodobenzene (**65**) for radiolabeling purposes in Sonogashira cross-coupling reactions [96]. The final aim of that work was the synthesis of novel fluorine-18 labeled steroids. On that account, a new synthesis procedure for the preparation of **65** was established using the respective iodonium salts **78a-c** which were treated with [^18^F]fluoride in a nucleophilic reaction as depicted in Scheme 32. To optimize the RCY of **65**, different counter ions (chloride, tosylate, triflate) of the respective iodonium salt as labeling precursor as well as several solvents and temperatures were tested. Irrespective of other reaction conditions, utilization of DMF as solvent always resulted in higher yields compared to acetonitrile or DMSO. Moreover, high reaction temperatures (>120 °C) seemed to be essential for an efficient thermal cleavage of **78a-c** by [^18^F]fluoride. No product formation was observed at a reaction temperature of 60 °C. Highest RCYs (up to 70%; determined by radio-TLC) of compound **65** were obtained when iodonium chloride **78a** was used in DMF under microwave conditions (5 min/120 watt) for the radiosynthesis of compound **65**. Upon completion of radiosynthesis, **65** was purified via solid phase extraction (SPE) followed by removal of the solvent. A comparison of the most commonly used methods for the preparation of **65** and **72** can be found in the literature [90].

Three precursors were radiolabeled with 4-[^18^F]fluoro-1-iodobenzene (**65**) using the Sonogashira cross-coupling. The reaction was performed in a sealed tube using THF as solvent and Et_3_N as base at 110 °C for 20 min. Upon addition of the terminal alkyne in the final reaction step, 85% of compound **65** were converted into the cyclopentyl carbinol **79**. Furthermore, radiolabeling of steroid precursors led to 65–88% and 34–64% conversion into steroids **80** and **81**, respectively.

Radiolabeling of nucleosides has become a highly attractive method over the past years due to their central role in biological systems. Several attempts were made to label nucleosides with the PET nuclides ^11^C, ^18^F, ^76^Br as well as ^124^I [97,98,99]. In the case of fluorine-18, two main strategies are described in most publications. The radionuclide is either introduced directly into the carbohydrate scaffold of a nucleoside possessing a good leaving group, or it is used to label a sugar which is subsequently linked to the respective nucleobase.

**Scheme 33:**
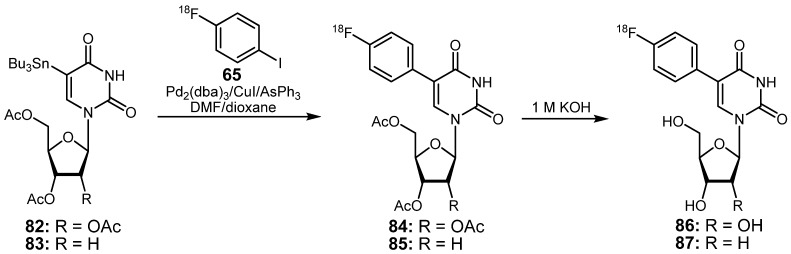
Preparation of uridine-based fluorine-18-labeled nucleosides.

Utilizing 4-[^18^F]fluoro-1-iodobenzene (**65**) as actual labeling agent, two novel ^18^F-labeled nucleosides **86** and **87** were prepared under mild conditions of the Stille cross-coupling (Scheme 33) [100]. For this purpose, a tributylstannyl group was incorporated into the pyrimidine moiety yielding uridine **82** and deoxyuridine **83**, respectively. The highest radiochemical yield (69%) of the reaction of **82** with **65** (which was synthesized from the respective iodonium salts [96]; *vide supra*) was obtained when the coupling reaction was carried out at 65 °C for 20 min in a DMF/dioxane (1:1) or THF/dioxane (1:1) mixture using Pd_2_(dba)_3_/CuI/AsPh_3_ as catalyst system. The radiofluorinated nucleoside **85** was synthesized in the same manner with a maximum RCY of 61% starting from the stannyl precursor **83** which was reacted with **65**. In both cases, the last step involved the cleavage of the acetyl protecting groups from the carbohydrate moiety of nucleosides **84** and **85**, which were removed quantitatively using a 1 M KOH solution at 65 °C for 10 min, yielding the final radiotracers **86** or **87** with 61% and 69% RCY (related to **65**), respectively, with a radiochemical purity of ≥95%.

The appropriate non-radioactive reference compounds were obtained in a similar way by a Stille cross-coupling using Pd(PPh_3_)_2_Cl_2_/CuI as catalyst in dioxane at 100 °C for 24 h followed by deprotection of the hydroxyl groups.

**Scheme 34:**
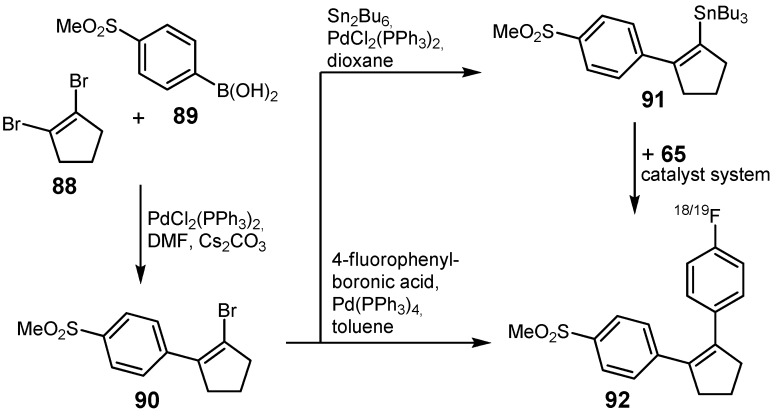
Preparation of the radiofluorinated COX-2 inhibitor **92** and the non-radioactive analog.

**Scheme 35:**
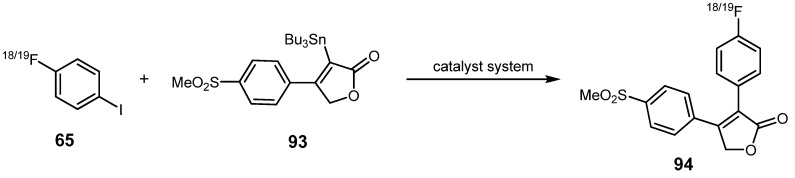
Preparation of the second COX-2 inhibitor **94** and its non-radiolabeled reference.

Cyclooxygenase (COX) enzymes play an important role in inflammatory and pain causing processes in general. Celecoxib and Rofecoxib represent two selective COX-2 inhibitors and are among the most widely used therapeutics for the treatment of pain and inflammation. Moreover, the COX-2 enzyme is overexpressed in many human cancer entities [101]. The group of Wuest *et al.* presented another approach using the Stille cross-coupling for radiofluorination purposes [102]. In this connection, two novel fluorine-18-labeled cyclooxygenase-2 inhibitors based on Rofecoxib were prepared with a slightly different core structure: One of these radiotracers comprehended a cyclopentene ring whereas the other contained a 3,4-substituted furanone unit. Different synthesis routes were investigated for the preparation of the labeled radiotracer **92** and the non-labeled analog based on the cyclopentene core as depicted in Scheme 34. Preparation of the second radiolabeled COX-2 inhibitor **94** as well as its non-labeled reference compound is depicted in Scheme 35.

Composition of the non-radioactive COX-2 inhibitor and its radiofluorinated counterpart **92** commenced with the preparation of methylsulfonylphenyl-substituted bromocyclopentene **90** which was synthesized from dibromocyclopentene **88** and boronic acid **89**.

Synthesis of **90** was modified as compared to the literature procedure and it was found that **90** could be prepared in a single step [103]. Improvements involved the utilization of DMF which appeared to be superior to the previously reported solvent system toluene/EtOH/2 M Na_2_CO_3_. Furthermore, a twofold excess of 1,2-dibromocyclopentene **88** compared to **89** was necessary to obtain **90** in a single step in 45% yield. Subsequent cross-coupling of **90** with 4-fluorophenyl boronic acid yielded the reference substance directly, whereas the preparation of fluorine-18-labeled compound **92** implied a two-step process: Initial reaction of **90** with Sn_2_Bu_6_ yielded **91**, which was subsequently converted into the desired radiotracer **92** utilizing 4-[^18^F]fluoro-1-iodobenzene (**65**). The radiolabeled COX-2 inhibitor **92** was obtained in 68% RCY relative to compound **65**.

**Scheme 36:**
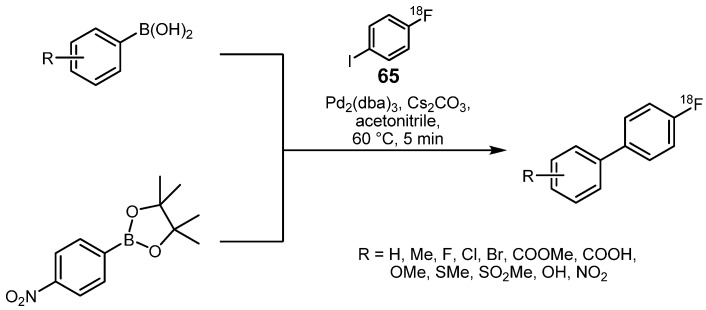
Model Suzuki labeling reactions.

Regarding the second COX-2 inhibitor, both **94** as well as the non-radiolabeled reference were prepared using the stannylated organotin compound **93** as depicted in Scheme 36. Optimized reaction conditions were applied for the synthesis of the ^18^F-labeled inhibitor **94** (Pd_2_(dba)_3_/P(*o*-Tol)_3_/CuI, DMF/toluene (1:1), 65 °C, 20 min) with 68% RCY based upon 4-[^18^F]fluoro-1-iodobenzene (**65**). The analogous non-radioactive compound was synthesized in 69% yield using the Pd(PPh_3_)_2_Cl_2_/CuI catalyst system in a toluene/ethanol (1:1) mixture at 50 °C overnight.

A further application using transition metal-mediated cross-coupling reactions for labeling purposes with fluorine-18 was demonstrated in 2006 with the implementation of the Suzuki cross-coupling [104]. Through this technique, a multitude of organoboron compounds were labeled with 4-[^18^F]fluoro-1-iodobenzene (**65**) to yield the respective biphenyl compounds in RCYs from 30 to 90% as demonstrated in Scheme 36. 

A wide variety of functional groups like esters, ethers, thioethers, halogens, alcohols and nitro derivatives were shown to be tolerable and compatible with the Suzuki cross-coupling. Excellent radiochemical yields greater than 80% within a synthesis time of just 5 min were achieved for most of the tested organoboron derivatives (except for: R = Br, Cl, COOH) with the Suzuki coupling of **65** using Pd_2_(dba)_3_ as mediator, Cs_2_CO_3_ as base and acetonitrile as solvent at 60 °C.

### 3.2. ^18^F-labeled compounds prepared via carbon-heteroatom cross-coupling reactions

**Scheme 37:**
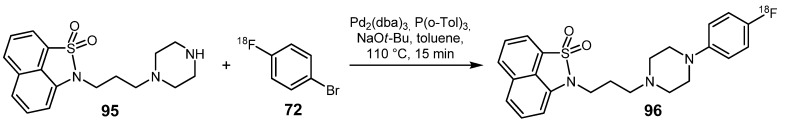
Radiolabeling with 1-bromo-4[^18^F]fluorobenzene **72** under Buchwald-Hartwig conditions.

Pd-mediated cross-couplings were also used for radiolabeling purposes which comprise the formation of carbon-heteroatom bonds. Marrière *et al.* were the first to use the synthetic approach [105] based on Buchwald-Hartwig conditions for the preparation of fanserin [^18^F]RP 62203, a 5-HT_2A_ serotonin receptor antagonist, as depicted in Scheme 37 [106]. Reaction of piperazine **95** with 1-bromo-4-[^18^F]fluorobenzene (**72**) in the presence of the catalyst system Pd_2_dba_3_/P(*o*-Tol)_3_ in toluene at 110 °C yielded radiolabeled fanserin **96** with 60% RCY after 15 min. Other methods the authors had attempted previously were either time consuming [107], too difficult to automate [108] or did not deliver sufficient specific activities [109].

**Scheme 38:**
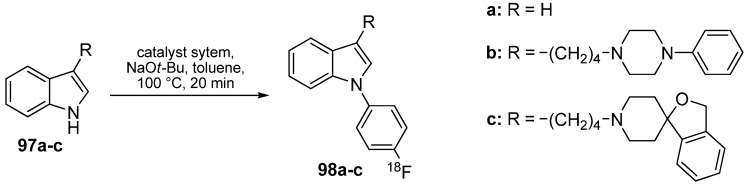
Preparation of model indole **98a** and σ_2_ receptor ligands **98b,c**.

The *N*-arylindole moiety represents a central structural motif found in pharmacologically important compounds such as angiotensin II-1 antagonists [110], melatonin receptor MT_1_ agonists [111], antipsychotic drugs [112] or selective σ_2_ receptor ligands [113,114]. Two examples (**98b,c**) of high affinity σ_2_ receptor ligands were successfully labeled using 4-[^18^F]fluoro-1-iodobenzene (**65**) [115]. For this purpose, **65** was prepared from the respective iodonium salts and reacted in a model reaction with 1*H*-indole (**97a**). Best labeling conditions were carried out using Pd_2_(dba)_3_ and 2-(dicyclohexyl-phosphino)-20-(*N*,*N*-dimethylamino)biphenyl as co-ligand in toluene and with NaO*t*-Bu as base to yield the radiofluorinated indole **98a** (70%) after 30 min synthesis time. Based on these results, two other inhibitors, as seen in Scheme 38, were prepared with RCYs of 91% for **98b** and 84% for **98c** (d.c.), respectively.

**Scheme 39:**
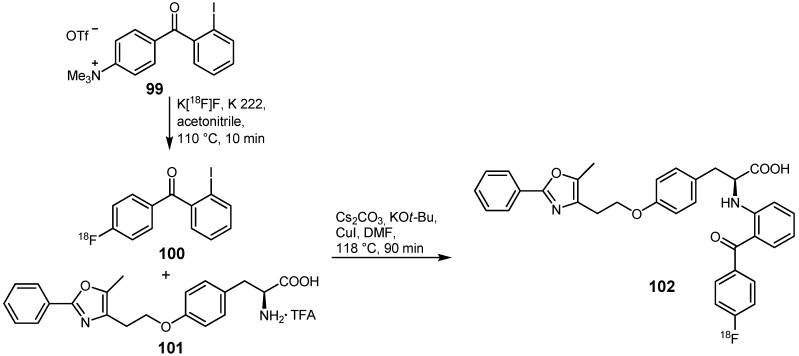
Preparation of **102** using Ullmann-type conditions.

Another example using the cross-coupling of nitrogen with carbon was demonstrated in 2009 [116] with the preparation of radiofluorinated farglitazar **102**, a compound for the imaging of peroxisome proliferator-activated receptor-γ ligands (PRARγ) [117,118]. Two synthesis routes for radiofluorination purposes were exemplified: On the one hand, direct labeling of the target molecule with Cs[^18^F]F was reported, whereas indirect labeling under Ullmann conditions was demonstrated on the other hand. 2-Iodo-4'-[^18^F]fluorobenzophenone (**100**) was prepared by means of the latter method with RCYs >97% by nucleophilic aromatic displacement of the trimethylammonium leaving group of precursor **99** (Scheme 39). The direct conversion of **100** with **101** via Ullmann condensation was unsuccessful, therefore the inorganic salts had to be removed prior to further reaction. After separation from by-products, CuI was added to **100**. In a second reaction vessel, **101** was dissolved in dry DMF and treated with a Cs_2_CO_3_/KO*t*-Bu mixture for 5 min. Afterwards, both mixtures were combined and heated to 118 °C for 90 min. The resulting green solution was purified via an HPLC system. The combination of Cs_2_CO_3_ and KO*t*-Bu instead of K_2_CO_3_ as base always achieved higher yields. In addition, the ratio between the starting material and the bases also influenced the yield. The optimal ratio between **99** + **101** and the base mixture was scrutinized to be 3:1, as stated by the authors.

**Scheme 40:**
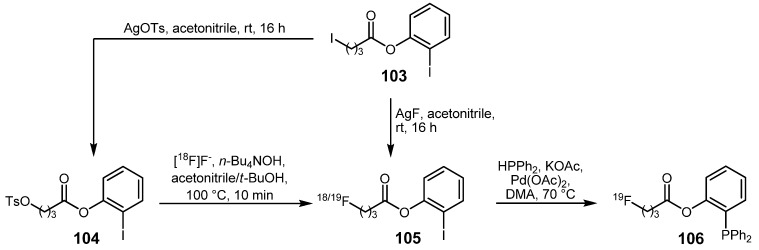
Synthetic approach to ^18^F-labeled phosphanes as precursors for Staudinger ligations.

A different approach for the synthesis of novel functionalized phosphanes was described in 2010 concerning the Pd-mediated phosphorus-carbon cross-coupling [119,120]. These phosphanes were introduced as starting material in labeling reactions using the traceless Staudinger ligation. For this purpose, *ω*-functionalized 2-iodophenyl carboxylic esters were prepared and labeled with fluorine-18 successfully (Scheme 40). Starting from 2-iodophenyl 4-iodobutanoate (**103**), tosylated compound **104** as well as the non-radioactive fluorine-19 compound **105** were prepared in high yields using the respective silver salts in acetonitrile at ambient temperature. Toslyate **104** served as starting material for the preparation of the radiolabeled fluorine-18 building block **105**. Optimal labeling conditions were evaluated by systematic variation of temperature and reaction time of which the latter seemed to have only little influence on the RCY of **105**. No radiofluorination product was obtained using DMF or DMSO and only low-yield conversion to **105** was observed when acetonitrile was used as solvent. An application of *n*-Bu_4_NOH instead of K_2_CO_3_ as base was important for a high radiochemical yield. Best conditions were obtained using a mixture of acetonitrile and *t*-BuOH (1:4) at 100 °C and a reaction time of 10 min with 58% conversion into the desired fluorine-18-containing 2-iodophenyl ester **105**. Unfortunately, the subsequent cross-coupling did not lead to the desired radiofluorinated phosphane **106** (Scheme 39), whereas the coupling reaction with the non-radiolabeled analog yielded the ^19^F-fluorinated phosphane **106** in 51% yield after 3 h.

## 4. Conclusions

Positron emission tomography (PET) is a sophisticated method for quantitative and noninvasive imaging of biological functions by monitoring the distribution of tracers labeled with positron emitters. A wide variety of labeled compounds has been developed as molecular imaging probes for the scrutiny of biochemical and physiological parameters. 

The present review summarized the implementation of transition metal cross-coupling reactions for labeling purposes with carbon-11 and fluorine-18 which has gained great interest for the preparation of radiotracers and radiopharmaceuticals for molecular imaging. Because of its functional group-tolerating character, mostly mild reaction conditions and short synthesis times, this kind of coupling reaction represents a valuable and multifunctional tool not only regarding the preparation of the precursor core. Especially in carbon-11 chemistry, time is an important criterion due to the short half- life of this isotope. As pointed out in this review, a multitude of cross-coupling reactions have been developed for the introduction of carbon-11, mostly in the form of [^11^C]methyl iodide or [^11^C]carbon monoxide, with high radiochemical yields and within short synthesis times. Advantageously, no secondary labeling building blocks have to be synthesized. Another benefit is that cross-coupling reactions normally yield by-products in very small amounts only; therefore side reactions can usually be neglected. However, in the case of labeling reactions, the carbon-11 species is only available in the nanomolar range. Thus, the prevention of side reactions as well as the purification of the labeled product become important to obtain the radiotracer in high radiochemical yields and with high specific activities.

Due to the non-physiological conditions (e.g. organic solvents, high temperatures) of most of the cross-coupling reactions, only small organic bioactive molecules could be labeled to date. No example of a labeled high molecular weight compound (peptide, protein or antibody) was found in the literature. However, due to the wide applicability, cross-coupling reactions have proven to be remarkable and valuable tools for the preparation of novel fluorine-18 and carbon-11-containing radiotracers based on small organic molecules. Consequently, recent trends in the evolution of metal-mediated cross coupling reactions for radiolabeling purposes will stimulate the positron emission tomography as powerful imaging technique with respect to clinical applications as well as drug research and development.

Cross-coupling reactions applied for radiofluorination purposes are of less importance due to the necessity to prepare primary radiofluorinated building blocks like the 4-[^18^F]fluorophenyl moiety. This represents an additional synthesis step in contrast to direct electrophilic and nucleophilic labeling procedures with fluorine-18. Thus, cross-coupling reactions are mostly applied for the preparation of the precursor core. In addition, special attention has to be paid to an application of cross-coupling reactions in the routinely large scale preparation of radiopharmaceuticals regarding GMP and GLP regulations. This will be a huge challenge in the future. 
